# Nano-Formulation of Ethambutol with Multifunctional Graphene Oxide and Magnetic Nanoparticles Retains Its Anti-Tubercular Activity with Prospects of Improving Chemotherapeutic Efficacy

**DOI:** 10.3390/molecules22101697

**Published:** 2017-10-12

**Authors:** Bullo Saifullah, Arundhati Maitra, Alina Chrzastek, Bullo Naeemullah, Sharida Fakurazi, Sanjib Bhakta, Mohd Zobir Hussein

**Affiliations:** 1 Mycobacteria Research Laboratory, Department of Biological Sciences, Institute of Structural and Molecular Biology (ISMB), Birkbeck, University of London, Malet Street, London WC1E 7HX, UK; bullosaif1@gmail.com (B.S.); arundhati.mrl@gmail.com (A.M.); alina.chrzastek@gmail.com (A.C.); s.bhakta@bbk.ac.uk (S.B.); 2 Material Synthesis and Characterization Laboratory, Institute of Advanced Technology (ITMA), Universiti Putra Malaysia (UPM), Serdang 43400, Selangor, Malaysia; 3 Laboratory for Vaccine and Immunotherapeutics, Institute of Biosciences, Universiti Putra Malaysia (UPM), Serdang 43400, Selangor, Malaysia; sharida@upm.edu.my; 4 Department of Neurology (Ward No. 18), Jinnah Postgraduate Medical Center/Jinnah Sindh Medical, University Karachi, Karachi 75510, Pakistan; bullonaeem@hotmail.com; 5 Department of Human Anatomy, Faculty of Medicine and Health Sciences, Universiti Putra Malaysia (UPM), Serdang 43400, Selangor, Malaysia

**Keywords:** Multifunctional nanocarrier, nanoformulation, ambutol, graphene oxide, iron oxide magnetite, tuberculosis

## Abstract

Tuberculosis (TB) is a dreadful bacterial disease, infecting millions of human and cattle every year worldwide. More than 50 years after its discovery, ethambutol continues to be an effective part of the World Health Organization’s recommended frontline chemotherapy against TB. However, the lengthy treatment regimens consisting of a cocktail of antibiotics affect patient compliance. There is an urgent need to improve the current therapy so as to reduce treatment duration and dosing frequency. In this study, we have designed a novel anti-TB multifunctional formulation by fabricating graphene oxide with iron oxide magnetite nanoparticles serving as a nano-carrier on to which ethambutol was successfully loaded. The designed nanoformulation was characterised using various analytical techniques. The release of ethambutol from anti-TB multifunctional nanoparticles formulation was found to be sustained over a significantly longer period of time in phosphate buffer saline solution at two physiological pH (7.4 and 4.8). Furthermore, the nano-formulation showed potent anti-tubercular activity while remaining non-toxic to the eukaryotic cells tested. The results of this in vitro evaluation of the newly designed nano-formulation endorse its further development in vivo.

## 1. Introduction

Tuberculosis (TB) is an infectious, bacterial disease caused by *Mycobacterium tuberculosis* (MTB) and it is a global health and economic concern, especially in the developing world [1,2]. On an average, it infects over 10 million people annually and kills over a million. Frontline chemotherapy for the treatment of drug-sensitive forms of the disease is a standardised six-month regimen of a cocktail of antibiotics, including the cell wall inhibitor-ethambutol (ETB). However, the protracted treatment regimen needed for frequent dosing and the hepatotoxicity caused as a result of the treatment complicates patient compliance, exacerbating the rising antimicrobial resistance crisis in TB.

Potentiating current therapy by altering the delivery and administration routes of these drugs is garnering attention in the international scientific communities. Nanomedicine is a rapidly advancing field in biomedical science where nanomaterials are designed and applied for theranostic (therapeutic and diagnosis) applications [3,4,5]. Multifunctional nanoparticles that combine physico-chemical properties of different nanomaterials resulting in improved characteristics are highly sought after as drug delivery systems [6]. Recently, a variety of nanomaterials-based antimicrobials agents have been developed [1,7,8,9]. Graphene oxide (GO) is a promising material for biomedical applications, especially in drug delivery systems because of its two dimensional (2D) nanosize providing a large surface area with different functional groups, such as hydroxyl, carbonyl, epoxides, and unsaturated benzene rings [10,11]. These functional groups allow different drugs, hydrophilic and hydrophobic, to be loaded on GO. Oxygenated functional groups enable drug loading via hydrogen bonding, electrostatic interaction, and unsaturated benzene rings help loading hydrophobic drugs via π-π interactions [10,12,13,14,15,16,17]. GO is getting increased attention for its applications in the design of new antibacterial therapy because of its tendency to induce oxidative stress by generating reactive oxygen species (ROS) and lipid peroxidation of the bacterial cell envelope [12]. GO disrupts bacterial physiological activity, its cell-wall and GO nano-sheets can trap bacteria better than graphene because of their oxygenated functional groups [12,18,19,20]. Because of the fascinating characteristics of GO, such as the ease of hydrophobic and hydrophilic drug loading, scalable preparation, better water dispersibility, sustained release, and its antibacterial properties make it an ideal candidate for drug delivery applications. Meanwhile, iron oxide magnetite nanoparticles (FeNPs) possess excellent, tailored surface properties, strong magnetic character and high biocompatibility, making them suitable for biomedical applications [4,21]. FeNPs have been widely applied in magnetic resonance imaging (MRI), hyperthermia, drug delivery, tissue repair, topical applications, bio-sensing, and bioanalysis [15,22,23,24,25,26]. Multifunctional nanoparticles based on GO and FeNPs are becoming increasingly popular as drug delivery agents because of their additive hyperthermia effect [27]. In this study, we have designed multifunctional nanoparticles formulation (nano-formulation) by fabricating graphene oxide with iron oxide magnetite nanoparticles (FeNPs-GO) loaded with the front-line anti-tubercular drug, ethambutol, resulting in anti-TB multifunctional nanoformulation (ETB-FeNPs-GO). The designed nano-formulation was characterized in detail using various analytical techniques and evaluated for biocompatibility and therapeutic efficacy.

## 2. Results

### 2.1. Physico-Chemical Characterization

#### 2.1.1. Powder X-ray Diffraction (XRD) of ETB-FeNPs-GO

**Figure 1:**
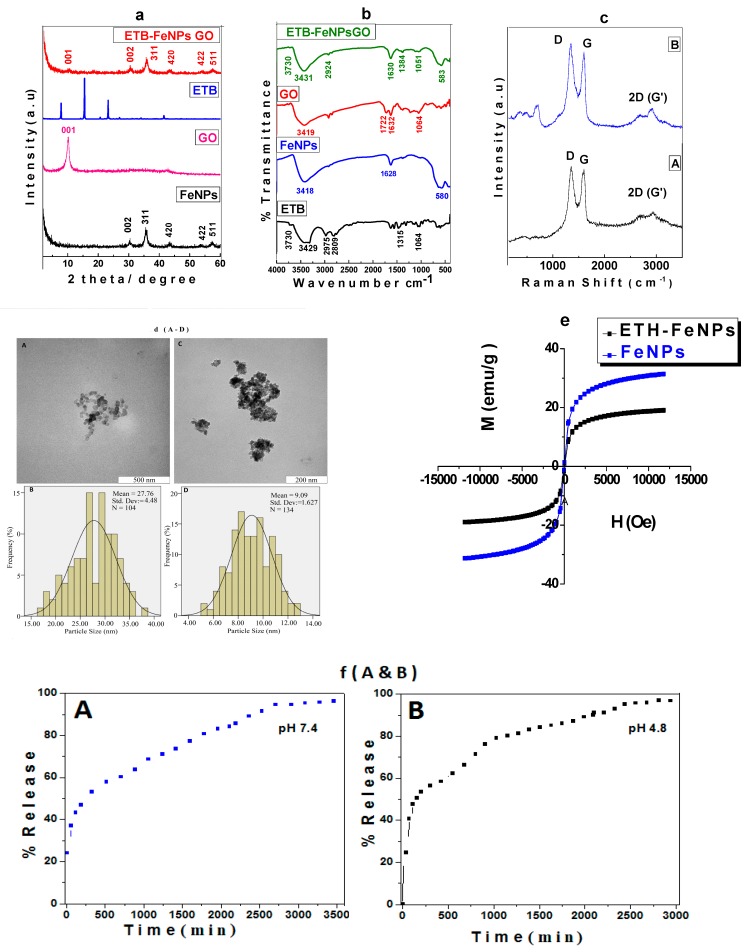
(**a**) X-ray diffraction patterns of iron oxide magnetite nanoparticles (FeNPs), free drug ethambutol (ETB), graphene oxide (GO), and anti-TB multifunctional nanoformulation (ETB-FeNPs-GO) (**b**) FTIR spectra of ETB, FeNPs, GO, and of ETB-FeNPs-GO; (**c**) Raman spectra of GO (A) and anti-TB multifunctional nanoformulation formulation ETB-FeNPs-GO (B); (**d**) Transmission electron micrograph (TEM) of FeNPs-GO (A) and their particle size distribution (B), TEM of anti-TB multifunctional nanoformulation (C) and their particles size distribution (D); (**e**) Magnetization plots of FeNP alone and multifunctional nanoformulation ETB-FeNPs-GO; (**f**) In vitro release profile of Ethambutol (ETB) from anti-TB multifunctional nanoformulation (ETB-FeNPs-GO) in PBS solutions of pH 7.4 (A) and pH 4.8 (B).

Figure 1a shows the x-ray diffraction (XRD) patterns of FeNPs, GO, free drug ETB and anti-TB multifunctional nanoformulation (ETB-FeNPs-GO). The FeNPs alone show five characteristic peaks because of (220), (311), (400), (422) and (511) planes at 2θ = 30.3°, 35.7°, 43.4°, 53.6° and 57.3°, respectively. These five peaks are an exact match to pure iron oxide magnetite nanoparticles showing a high purity of the designed FeNPs having cubic inverse spinel structure [28]. The free drug ETB showed three crystalline intense peaks at 2θ of 7.86°, 15.48° and 23.25° due to the crystalline characteristics of organic molecule [14]. GO showed a characteristic peak at about 2θ = 10.0° due to the (001) planes and no peak is observed due to starting material graphite which usually appears at 2θ = 26.27° and the absence of graphite peaks indicates the high purity of GO [29]. The anti-TB multifunctional nanoformulation (ETB-FeNPs-GO) showed a diffraction peak at 2θ = 10.0° due to the (001) planes of GO as well as the characteristic peaks of FeNPs due to (220), (311), (400), (422) and (511) planes at 2θ = 30.3°, 35.7°, 43.4°, 53.6° and 57.3°, respectively. The presence of these five characteristic peaks of iron oxide magnetite nanoparticles and typical peak of GO at about 2θ = 10.0° in the multifunctional nanoformulation (ETB-FeNPs-GO) confirms the successful formation. The drug ETB did not show any peak in the ETB-FeNPs-GO, which is a common phenomenon that has been reported previously [24,30]. The presence of ETB loading on FeNPs-GO was confirmed by other complimentary techniques like FTIR, HPLC, and UV/Vis spectroscopy discussed in detail in the later sections.

#### 2.1.2. Fourier Transformed Infrared (FTIR) Spectroscopic Analysis

Figure 1b represents the FTIR spectra of free drug ETB, FeNPs, GO, and anti-TB multifunctional nanoformulation (ETB-FeNPs-GO). The free drug ETB showed characteristic absorption bands of its functional groups, namely stretching bands for N-H, O-H, C-H, C-N, and C-O at 3739 cm^−1^, 3419 cm^−1^, 2975 cm^−1^, 2809 cm^−1^, and 1060 cm^−1^, respectively [31,32]. The iron oxide magnetite nanoparticles showed typical peaks of O-H, F-O stretching bands at 3419 cm^−1^ and 572 cm^−1^, respectively [24]. Different functional groups, bands peaks associated with GO such as O-H, carbonyl (C=O), C=C, alkoxy and epoxides groups were observed at 3429 cm^−1^, 1722 cm^−1^, 1629 cm^−1^ and 1064 cm^−1^, respectively [14,29,33]. The anti-TB multifunctional nanoformulation showed the functional groups bands of ETB, FeNPs, and GO with slight shifts. The presence of bands belonging to the functional group of ETB, FeNPs, and GO indicate successful formation anti-TB multifunctional nanoformulation (ETB-FeNPs-GO). Table 1 shows the assignment of each individual band of the sample.

#### 2.1.3. Raman Spectroscopy Analysis

Raman spectroscopy is a useful technique for the analysis of defects, disorder, and changes in the structure of carbon based material due to chemical modifications/reactions [34]. Figure 1c shows the Raman spectra of GO and ETB-FeNPs-GO. In the Raman spectrum of GO, two peaks, D and G, were observed due to the characteristics graphitic nature. The D band is due to the disorder-induced mode, and the G-band is due to the graphitic-like mode that appeared at 1349 and 1557 cm^−1^, respectively [35,36,37]. The presence of the D and G bands in the multifunctional nanoformulation with almost similar intensity, as shown by GO Raman bands, indicates the presence of GO in the ETB-FeNPs-GO. The ratio of ID over IG for GO alone and ETB-FeNPs-GO was found to be 1.0 when compared to the graphite intensity ratio of ID/IG = 0.833, which is in agreement with previous studies [35].

#### 2.1.4. HPLC and ICP Analysis

The quantification of iron (Fe) content and ETB loading was determined using inductively coupled plasma (ICP) and high performance liquid chromatography (HPLC) analysis, respectively. For iron content quantification, various standards of Fe i.e., 25 ppm, 50 ppm, 100 ppm, 200 ppm, and 250 ppm were analysed by ICP, the calibration curve was plotted, and r^2^ was found to be 0.9987. The amount of iron (Fe) in the multifunctional nanoformulation ETB-FeNPs-GO was found to be 62.30% (1.11 moles). The drug (ETB) loading in the designed multifunctional nanoformulation (ETB-FENPs-GO) was determined by HPLC analysis. For the calibration curve, different standard concentrations of ETB were prepared e.g., 25 ppm, 50 ppm, 100 ppm, 150 ppm, 200 ppm, and r^2^ was determined to be 0.9980 (graph of calibration curve is given in Supplementary Material Figure S1). The loading of the ETB in anti-TB multifunctional nanoformulation (ETB-FeNPs-GO) was found to be 33.83% (0.166 moles). The empirical formula of the designed anti-TB multifunctional nanoformulation (ETB-FeNPs-GO) was determined to be [(C_10_H_24_N_2_O_2_)_0.166_ (Fe_3_O_4_)_1.11_ (GO)_(x)_].

#### 2.1.5. Transmission Electron Microscopic (TEM) Analysis

Figure 1d (A–D) shows the transmission electron micrographs of multifunctional nanocarrier alone FeNPs-GO (A), its particle size distribution of FeNPs-GO (B), the anti-TB multifunctional nanoformulation ETB-FeNPs-GO (C), and its particle size distribution of ETB-FeNPs-GO (D), respectively. The shape of multifunctional nanocarrier FeNPs-GO was found to be roughly circular. The particle size distribution was measured using image processing software (UTHSCSA Image Tool for Windows Version 3.00) by randomly selecting 104 particles (N), and it was determined to be of 27.76 nm ± 4.458 nm. The shape of ETB-FeNPs-GO was also found to be roughly circular with the particles being agglomerated. The particle size of the ETB-FeNPs-GO was determined by randomly selecting 134 particles (N), and it was found to be 9.09 nm ± 1.627 nm. The small particle size of the ETB-FeNPs-GO compared to empty carrier FeNPs-GO may possibly be attributed to the extra stirring during the drug loading process and the smaller size of drug loaded when compared to the empty carrier has also been reported previously [20,38].

### 2.2. Magnetic Properties

Magnetic properties of the FeNPs and of the ETB-FeNPs-GO were analysed using Vibrating Sample Magnetometry (VSM) analysis. Figure 1e shows the VSM hysteresis loops as a function of the magnetic field at room temperature. For iron oxide magnetite nanoparticles, saturation magnetization (Ms) was determined to be 31.36 emu/g with a remnant magnetization (Mr) of 0.78 emu/g. For anti-TB multifunctional nanoformulation, ETB-FeNPs-GO saturation magnetization was found to be 19.02 emu/g with remnant magnetization (Mr) 0.70 emu/g. The VSM analysis data revealed that both samples possess superparamagnetic behaviour because the samples did not show any magnetism in the absence of magnetic field. The superparamagnetic nature of the multifunctional nanoformulation (ETB-FeNPs-GO) makes it more suitable for the target delivery drug and other biomedical application with additive hyperthermia properties. These magnetic nanoparticles have the tendency to get hotter when external magnetic field is applied and have been used in killing/eradication bacteria and cancerous cells by the hot FeNPs, called killing by magnetic hyperthermia [39,40].

#### 2.2.1. In vitro Drug Release Study

Figure 1f shows the in vitro release profile of ETB from anti-TB multifunctional nanoformulation in phosphate buffered saline (PBS) solution of pH 7.4 (A) and in PBS solution of pH 4.8 (B). Under both conditions of the PBS solution, the initial (~40%) releases were relatively faster when compared to the later 60% release. This faster release can be attributed to the less tightly bonded/adsorbed drug molecules within multifunctional nanocarrier FeNPs-GO and the later stage, sustained release can be attributed to the tightly bound drug molecules. The total time for 100% release of the drug was determined to be about 3000 min (50 h) under both conditions. The free drug ETB under similar conditions took 10 min for 100% release, as we have previously reported [41]. This suggests that multifunctional nanoparticles formulation ETB-FeNPs-GO sustained the release for much longer time when compared to free drug ETB. This sustained release characteristic has implications in improving the bioavailability of the drug.

#### 2.2.2. Nano-Formulation Shows Antimycobacterial Activity

**Figure 2:**
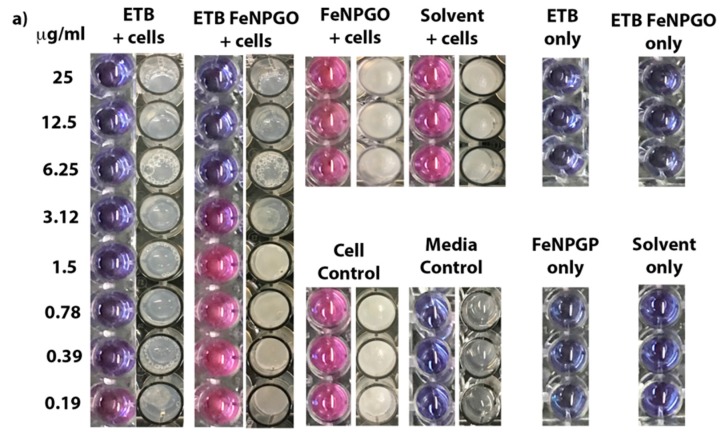

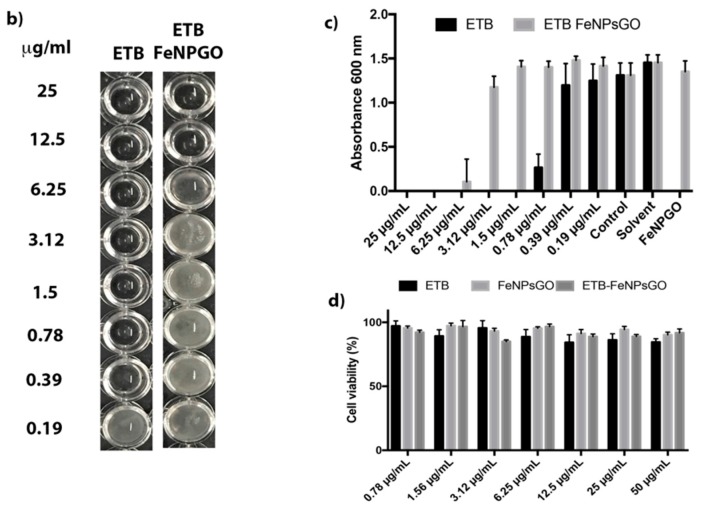
(**a**) REMA and SPOTi results based on ETBambutol (ETB) and multifunctional nanoformulation ETB-FeNPs-GO, tested on *M. smegmatis*; (**b**) Complete biofilm inhibition in 96 well plate results based on ETB and ETB-FeNPs-GO tested on *M. smegmatis* according to their MIC; (**c**) Crystal violet quantification of concentration-dependent inhibition of biofilmsby ETB and ETB-FeNPs-GO; (**d**) Cell viability determined using MTT for the ETB, FeNPs-GO, and ETB-FeNPs-GO.

Minimum inhibitory concentrations (MIC) of free drug ETB and ETB-FeNPs-GO was determined against *M. smegmatis* using REMA and modified SPOTi assay, as shown in Table 2 and Figure 2a. The loading percentage of ETB on FeNPs-GO as determined by HPLC is 33.83%. Taking this into account, the effective MIC of ETB-FeNPs-GO is 2.1 µg/mL.

#### 2.2.3. Biofilm Inhibition by ETB-FeNPs-GO

ETB-FeNPs-GO was found to inhibit the formation of a continuous biofilm at the air-liquid interface at the MIC for planktonic cells (effective concentration 2.1 µg/mL). Complete inhibition of all cell growth is seen at a concentration that is two-fold higher than the MIC (effective concentration 2.1 µg/mL). ETB alone inhibits biofilm formation in *M. smegmatis* at its MIC of 0.39 µg/mL, apparently, resulting in better inhibition in vitro than the nanoformulation (shown in Figure 2b,c) reasonably, due to the sustained release of the drug.

#### 2.2.4. Cytotoxicity Studies

In vitro cytoxicity profile of free drug ETB, nanocarrier FeNPs-GO, and multifunctional nanoformulation (ETB-FeNPs-GO) was evaluated on eukaryotic 3T3 cells using a range of concentrations (i.e., 0.78–50 ug/mL) of all the samples. Untreated cells were taken as standard. Cells were incubated with the samples for 72 h and cell viability was determined using MTT assay. All of the samples were found to be highly biocompatible as the cell viability was found to be more than 80% after 72 h treatment period as shown in Figure 2d.

## 3. Discussion

Here, we report the design of a multifunctional anti-tuberculosis nanoformulation by the fabrication of iron oxide magnetite (FeNPs) and graphene oxide. The first line anti-TB drug, ethambutol, was successfully loaded onto the designed multifunctional nanocarrier (FeNPs-GO). Release studies conducted in human body-simulated PBS solution of human blood pH 7.4 and intracellular lysosomal pH 4.8 found the sustained release of up to 50 h when compared to free drug ETB, which took 10 min for 100% release under similar conditions. The prolonged sustained release property of designed formulation has the potential to reduce the dosing frequency for treatment. Additionally, multifunctional anti-TB nanoformulation (ETB-FeNPs-GO) was found to be highly biocompatible with cell viability of about 90% when 3T3 fibroblast cells were exposed to 50 μg/mL of the formulation for up to 72 h. The anti-mycobacterial activity of ETB in the formulation is lower than that of the free drug. However, incubation periods of 24 h would not have been enough to release the entire drug-load bound to the nanoformulation. The designed formulation shows potent antimycobacterial activity, and most importantly, was found to maintain its superparamagnetic character. The superparamagnetic nature can be further investigated for its application in the hyperthermic killing of pathogenic bacteria. We believe this work to be a step forward in making the chemotherapy of TB patient-friendly with enhanced bioavailability and therapeutic effects achieved through reduced dosing frequency.

## 4. Materials and Methods

### 4.1. Materials

Pure drug Ethambutol dihydrochloride (99% purity), hydrogen peroxide (H_2_O_2_), potassium permanganate (KMnO_4_), sulfuric acid (H_2_SO_4_, 98%), graphite flakes with 109 meshes, phosphate-buffered saline, and phosphoric acid (H_3_PO_4_), were purchased from Sigma Aldrich (St. Louis, MO, USA) and were used without further purification. Sodium hydroxide, diethyl ether, and hydrochloric acid (HCl, 37%) were purchased from Friedemann Schmidt (Parkwood, WA, USA). The 3T3 fibroblast cell lines were purchased from the American Tissue Culture Collection (Manassas, VA, USA) and deionized water was used in all experiments.

### 4.2. Instrumentations

Shimadzu XRD-6000 Diffractometer (Shimadzu Corporation, Tokyo, Japan) was used for X-ray diffraction (XRD) studies with CuKα radiation at 30 kV and 30 mA. A Perkin-Elmer 100 series spectrophotometer Fourier transform infrared (FTIR) (Waltham, MA, USA) was applied for the functional group analysis and spectra were recorded by the direct sample method. For the optical and in vitro release studies a Perkin Elmer Lambda 35 ultraviolet-visible spectrophotometer (Waltham, MA, USA) was used. The Raman spectra were recorded with excitation wavelength (598 nm) using Raman spectrometer Witec Model: Alpha 300R (Witec, Ulm, Germany). The drug loading was quantified using Sykam high performance liquid chromatography (HPLC) system (SYKAM GmbH, Eresing, Germany) was used with the a Sykam S3250 UV/Vis detector auto injector Sykam 5300, and the Sykam quaternary pump system 5300 made in Germany, with a Zorbax Rx-Sil column of 4.6 × 150 mm, with 5 μm particle size (Agilent Technologies, Santa Clara, CA, USA).

### 4.3. Synthesis of Graphene Oxide

Graphene oxide was synthesized via oxidative exfoliation by an improved method [29]. In short, concentrated H_2_SO_4_ (360 mL) and concentrated H_3_PO_4_ (40 mL) were added to a mixture of graphite powder (3 g) and KMnO4 (18 g), and the solution was kept on stirring under constant heating at 50 °C for 12 h. The sample was cooled down to room temperature and poured onto cubes of ice (400 g) containing 3 mL of hydrogen peroxide (H_2_O_2_). Synthesized GO was subjected to work up by successive washing with water (200 mL), hydrochloric acid (HCl) (200 mL), ethanol (200 mL), followed coagulation with diethyl ether (200 mL), and the final product was dried in an oven at 40 °C [29].

### 4.4. Synthesis of Iron Oxide Magnetite Nanoparticles Fe_3_O_4_ (FeNPs)

Iron oxide magnetite nanoparticles were synthesized using the previously reported method [24]. In short, in 80 mL aqueous solution of Ferric chloride hexahydrate (FeCl_3_·6H_2_O) 0.99 g plus Ferrous chloride tetra hydrate (FeCl_2_·4H_2_O) (2.43 g), and a few drops of 5 M NaOH solution were added under vigorous stirring till the pH rose to 11 and black precipitates were formed. The synthesized product was subjected to ultra-sonication for an hour (at ultrasonic irradiation of 20 kHz), and then subjected to work up with thorough washing by deionized water, dried in oven for 24 h at 70 °C, and the product was ground to powder [24].

### 4.5. Synthesis of Multifunctional Nanoparticles FeNPs-GO and Loading of Ethambutol (ETB)

The iron oxide magnetite nanoparticles (FeNPs) product was added to 50 mL of 1% GO aqueous solution and the sample was stirred for 24 h at room temperature. The obtained product was washed, centrifuged, and dried at 40 °C, and was ground to powder. For drug loading, 0.25 g of multifunctional nanoparticles (FeNPs-GO) were added to 1% aqueous solution of ETB followed by 24 h stirring. After that, the sample was washed with water followed by centrifugation, dried in an oven at 40 °C and the resulting sample contained anti-TB multifunctional nanoparticles nanoformulation (referred to as nano-formulation in rest of the manuscript).

### 4.6. Sustained Release of Nano-Formulation In Vitro

In vitro release of ETB from the nanoformulation was monitored in phosphate buffer saline (PBS), simulating human blood (pH 7.4) and lysosomal (pH 4.8) conditions. Approximately 4 mg of the ETB-FeNPs-GO nano-formulation was placed in 3.5 mL of each PBS solution separately and release behaviour of ETB was recorded using UV/Vis spectrophotometer using lambda max of ETB 192 nm.

### 4.7. High Performance Liquid Chromatography (HPLC) Analysis for ETB Loading

HPLC analysis was performed for drug loading quantification of the ETB in designed nano-formulation (ETB-FeNPs-GO) using previous method [42]. In short, the solvent system used was acetonitrile (solvent A) and 15 mmol/L potassium dihydrogen phosphate buffer with pH adjusted to 4.0 ± 0.1 (solvent B). The solvent systems were made in the ratio of 89:11 (A:B) with a flow rate of 1 mL/min at 25 °C and UV detector was used with set wavelength of 190 nm. For calibration curve different standard concentrations of ETB i.e., 25 ppm, 50 ppm, 100 ppm, 150 ppm, and 200 ppm were analyzed by HPLC and r^2^ was found to be 0.9980. The multifunctional nanoformulation 10 mg was put in 10 mL of 1 molar of PBS buffer and sonicated for 1 h and sample was kept in the same buffer for 15 days to ensure 100% percent release of the drug. After that, the sample was filtered using 2 μL filter and then transferred to the HPLC injection vial for HPLC analysis, and three different samples of nanoformulation (ETB-FeNPs-GO) were prepared in the similar way. Samples were analyzed using above HPLC protocol after running the ETB calibration standards. The calibration curve for the standards is given in the Supplementary Material Figure S1.

### 4.8 Biological Evaluation

#### 4.8.1. MTT Assay for In Vitro Cytotoxicity Studies

The MTT [3-(4,5-dimylthiazol-2-yl)-2,5-diphenyltetrazolium bromide] assay was utilized for the cytotoxicity evaluation of the nanocarrier (FeNPs-GO), free drug ETB, and anti-TB nano-formulation (ETB-FeNPs-GO). Cells with a density of 1.0 × 10^5^ cells per well were seeded in 100 μL of cell culture medium. After one day of the cells incubation, cells were treated with all the above samples various (0.78 to 50 μg/mL) in triplicates and incubated for 72 h. After 72 h, the MTT solution concentration of 5 mg/mL was added to each well then incubated further for 4 h. After 4 h 100 μL of dimethyl sulfoxide was added to each well for the dissolution of dark blue formazan crystals resulted by the reduction of tetrazolium by living cells. The living cells were quantified by microplate enzyme-linked immunosorbent assay reader (ELx800, BioTek Instruments, Winooski, VT, USA) choosing wavelength of 570 nm and 630 nm [43]. Cell viability data presented in the percentage values are in comparison to the untreated cells (standard) under the same experimental conditions. The experiments were carried out in triplicate for each sample and results are presented with mean ± standard deviation.

#### 4.8.2. Resazurin Microtiter Assay (REMA)

The minimum inhibitory concentration (MIC) was established by the drug susceptibility assay Resazurin Microtiter Assay (REMA) [44]. In brief, 2 µL of the initial drug/nanoformulation stock of concentration 100 µg/mL was added to 198 µL of media in a microtiter plate, and 100 µL of this was used to serially dilute (two-fold) the drug along the columns of the plate. Mycobacterial culture was grown until early-logarithmic phase (OD_600_~1.00) and 100 µL of 10^−3^ dilution of the cell suspension was added to each well [45]. *M. smegmatis* was incubated with the compounds for 24 h at 37 °C. Solvent (DMSO), carrier (FeNPs-GO), drug-free, and cell-free controls were set up. After incubation, 30 µL of resazurin dye mixture (1:1 (*v*/*v*) of 0.01% (*w*/*v*) resazurin and 20% (*v*/*v*) Tween-80%) was added to each well and was incubated for a further 6–9 h. A change from blue to pink indicates the growth of bacteria. The MIC was determined by the lowest concentration of the compound that prevents the colour change. The experiment was performed in triplicate.

#### 4.8.3. Modified SPOTi Assay

This assay is based on SPOTi assay [46,47,48,49]. *M. smegmatis* cells were grown (37 °C, 180 rpm) to logarithmic growth phase (OD_600_~1.00) and 100 µL of 10^−3^ dilution of the bacterial cell suspension (1 × 10^5^ CFU) was incubated with a range of serially diluted concentrations of the drug and its nanoformulations (25–0.1 µg/mL) in a 96 well microtitre plate. Solvent (DMSO), carrier (FeNPs-GO), drug-free and bacilli-free controls were set up. The plate was incubated for 24 h at 37 °C, shaking at 150 rpm for maintaining a uniform suspension of cells and the formulation in solution. After the end of incubation, 2 µL of the cell suspension was spotted onto microtiter plate with each well containing 200 µL of M7H10 media (BD Biosciences, Swindon, UK) supplemented with 0.02% (*v*/*v*) glycerol and 10% Oleic acid-Albumin-Dextrose-Catalase (OADC, BD Biosciences, UK). The plate was incubated at 37 °C for three days until visible spots of bacterial growth appeared in the wells inoculated with cell suspension from the drug-free controls. The experiment was performed in triplicate.

#### 4.8.4. Biofilm Inhibition Assay

Biofilm growth and inhibition assay was prepared in 96 well plates (Corning, polystyrene, non-treated) [50]. *M. smegmatis* cells were grown to late-logarithmic phase (OD_600_ ~4) and 10 µL of the cell culture was added to each well containing up to 200 µL of Sauton’s media (0.5 g KH_2_PO_4_, 0.5 g MgSO_4_, 4.0 g l-asparagine, 0.05 g ferric ammonium citrate, 2.0 g citric acid, 60 mL glycerol dissolved in 900 mL dH_2_O and pH adjusted to 7) containing the drug or formulation in a range of concentrations between 25–0.19 µg/mL. Solvent (DMSO), carrier (FeGO), drug-free, and cell-free controls were set up. The plates were left in the incubator for five days at 37 °C, static conditions. After the given incubation time, the media containing planktonic cells was aspirated and the plate was left in a hot oven at 50 °C for 10 min to allow for the biofilm to dry. Crystal violet (0.1% *v*/*v*) was added to each well and left to incubate for 30 min at room temperature. Lastly, 30% acetic acid was added to solubilise the biofilm for 30 min at room temperature. The content of each well was transferred to a new 96 well plate and the OD_600_ was read using a plate reader Biotek (Synergy, Perth, Australia). The experiment was carried out in triplicate. Solvent (DMSO), carrier (FeNPs-GO), drug-free, and bacilli-free controls were set up.

## Figures and Tables

**Table 1:** FTIR assignments of absorption bands of free ETB, GO, FeNPs, and multifunctional nanoformulation (ETB-FeNPs-GO).

Assignments	Free ETB	FeNPs	GO	ETB-FeNPsGO
N-H Stretching	3739 cm^−1^	-	-	3730 cm^−1^
O-H Stretching	3419 cm^−1^	3419 cm^−1^	3429 cm^−1^	3431 cm^−1^
C-H Stretching	2975 cm^−1^	-	2975 & 2809 cm^−1^	2924 cm^−1^
C=O, C=C stretching	-	-	1722 & 1629 cm^−1^	1630 cm^−1^
C-N Stretching	1315 cm^−1^	-	-	1384 cm^−1^
C-O Stretching	1060 cm^−1^	1034 cm^−1^	~1064 cm^−1^	1051 cm^−1^
F-O stretching	-	575 cm^−1^	-	583 cm^−1^

Abbreviations in Table 1: ETB (Ethambutol), FeNPs (iron oxide magnetite nanoparticles), anti-TB multifunctional nanoformulation (ETB-FeNPs-GO).

**Table 2:** The minimum inhibitory concentration of the compounds tested based on the liquid culture Resazurin Microtiter Assay (REMA) assay with *M. smegmatis.*

Compound	REMA		Modified SPOTi	
	Observed MIC (µg/mL)	Effective MIC (µg/mL)	Observed MIC (µg/mL)	Effective MIC (µg/mL)
ETB	0.39	0.39	0.39	0.39
ETB-FeNPs-GO	6.25	2.1	6.25	2.1

## Supplementary Materials

Supplementary materials are available online.
