# Supplementary material for: Nano-Formulation of Ethambutol with Multifunctional Graphene Oxide and Magnetic Nanoparticles Retains Its Anti-Tubercular Activity with Prospects of Improving Chemotherapeutic Efficacy
Source: Molecules. 2017 Oct 12;22(10):1697. doi: 10.3390/molecules22101697 (PMC6151652; doi:10.3390/molecules22101697)
Supplement: Supplementary file 1 [file molecules-22-01697-s001.pdf]

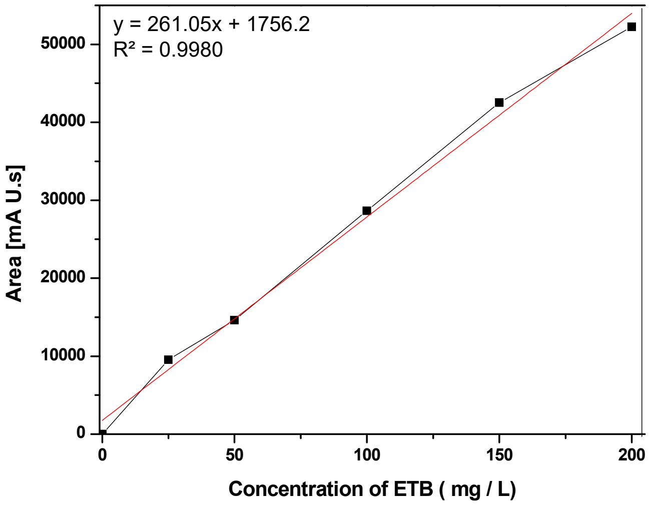

Figure S1. Calibration curve of ETB determined by using HPLC with different standard concentrations of 0 mg/L, 25 mg/L, 50 mg/L, 100mg/L, 150 mg/L, and 200 mg/L.

Notes: The red line shows linear fitting of calibration curve and the black line shows the experimental data.
